# Thiol–Ene Click Cross-linking of Starch Oleate Films for Enhanced Properties

**DOI:** 10.1021/acs.biomac.3c00507

**Published:** 2023-11-07

**Authors:** Laura Boetje, Xiaohong Lan, Jur van Dijken, Gerbrich Kaastra, Michael Polhuis, Katja Loos

**Affiliations:** † Macromolecular Chemistry & New Polymeric Materials, Zernike Institute for Advanced Materials, University of Groningen, Nijenbogh 4, 9747AG Groningen, The Netherlands; ‡ Hogeschool Van Hall Larenstein, 8934 CJLeeuwarden, The Netherlands; § Royal Avebe U.A., Zernikelaan 8, 9747AA Groningen, The Netherlands

## Abstract

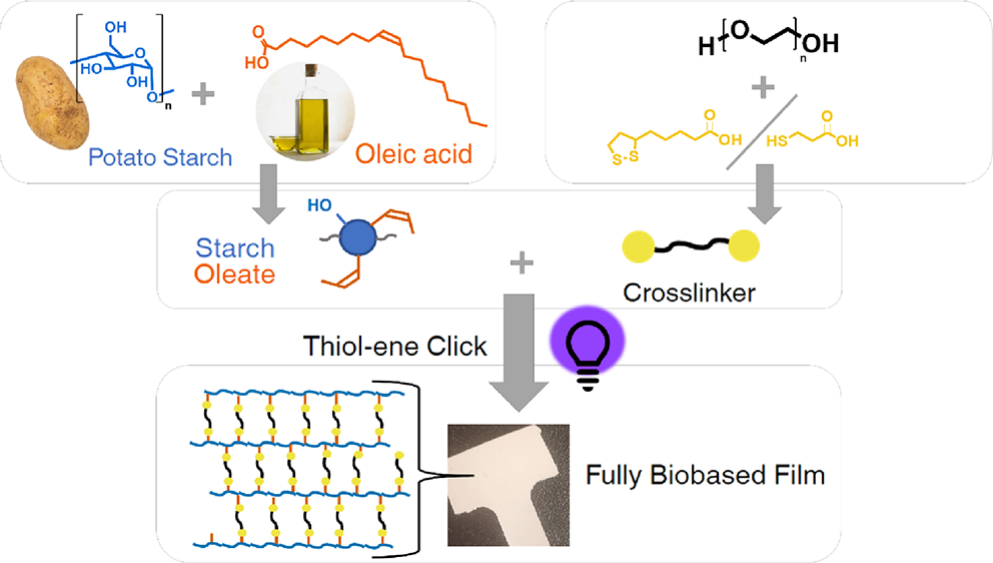

Biobased films were synthesized from starch oleate (DS = 2.2) cross-linked with polyethylene glycol with *M*_n_ = 2000 and 1000 g · mol^–1^, and ethylene glycol, all of which were esterified with either lipoic acid (LA) or 3-mercaptopropionic acid (MPA). Cross-linking was achieved through a UV-initiated thiol-ene click, and confirmed by Fourier transform infrared spectroscopy and rheometry. The films exhibit higher degradation temperatures, and an increased degree of crystallinity as cross-linker length increased. The introduction of MPA-based cross-linkers resulted in hydrophilic films, while the contact angle was barely affected by the addition of LA-based cross-linkers. A reduction in maximum strength upon introducing the cross-linkers was observed, while an increase in elongation was observed for most of the LA-based cross-linkers. Our results demonstrate the potential for tuning the mechanical and thermal properties of starch-based films through the cross-linker choice, with some formulations exhibiting increased flexibility that may be well suited for packaging applications.

## 1 Introduction

Petroleum-based resources have long been the materials of choice for numerous industries, but due to their finite nature and contribution to global warming, there is an increasing demand for sustainable and biobased alternatives. Starch, being one of the most abundant carbohydrates, is low in cost and nontoxic, making it an excellent candidate as a biobased alternative to potentially replace petroleum as a source of plastic materials.^1^

Our previous research has focused on the efficient synthesis of fatty acid starch esters,^2^ followed up by an investigation into the properties of the corresponding films cast from solutions of these starch esters.^3^ The tensile properties of these films were subsequently further enhanced via cross-linking of the starch oleate (SO) through the unsaturated bonds, using both heat curing and UV curing in the presence of suitable photoinitiators.^3,4^

In addition to direct cross-linking of the oleate double bonds, the double bonds are also susceptible to thiol addition via a thiol–ene click reaction.^5−7^ This reaction can be initiated by UV light and has the advantage of being fast, highly efficient, and hardly influenced by the presence of oxygen or water.^8^ Several studies have already shown the addition of thiol-containing products to vegetable oils via a thiol–ene click reaction.^9−11^ However, SO films cross-linked via a thiol–ene click reaction have not been studied before.

There exists a wide variety of different cross-linkers, including polyethylene glycol (PEG), a commercial biobased polymer available in a wide range of molecular weights.^12^ Cross-linking with highly flexible PEG can influence the thermal and mechanical properties of various materials, and an increased molecular weight of PEG can increase maximum elongation at break for the material.^13,14^ Moreover, since PEG polymer chains contain hydroxy groups at the termini, there exists the possibility of their esterification with several biobased thiol-containing acids, which in turn opens up the possibility of their application in thiol–ene click reactions.^15,16^

**Scheme 1:**
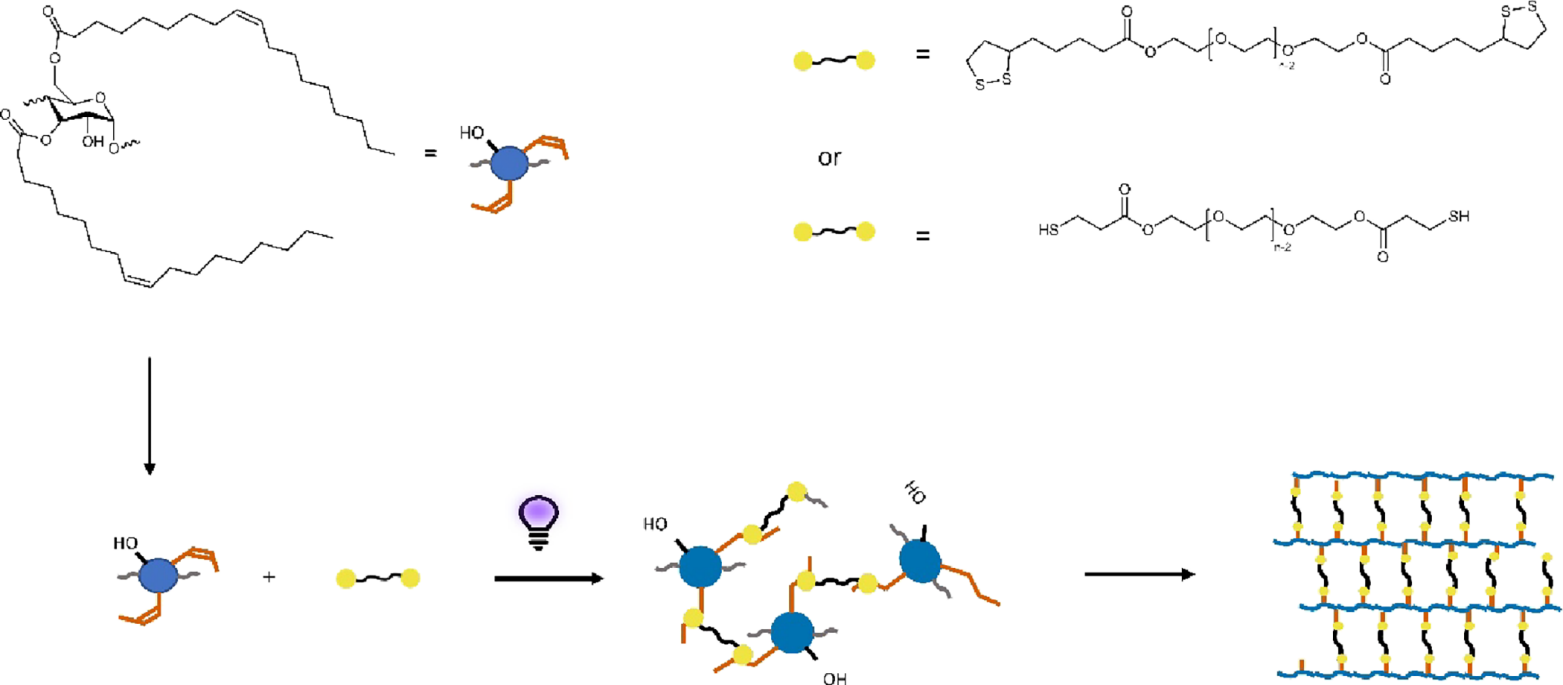
The Cross-linking Process of SO with Esterified PEG

In this work, a series of PEGs with varying molecular weights were esterified with biobased 3-mercaptopropionic acid (MPA) and lipoic acid (LA). Several studies have already shown the successful esterification of PEG with MPA and their applicability to a thiol–ene click reaction in, for example, hydrogel formation.^15,16^ We included LA in the study since it contains a disulfide that can participate in cross-linking via four S atoms instead of two. The cross-linking of MPA and LA with the double bond of SO double bonds is illustrated in Scheme 1.

This paper investigates SO photocured with PEG-modified cross-linker series with varying molecular weights and end groups. With the incorporation of an appropriate amount of PEG, the thermal and mechanical behavior of the films could be tuned without compromising their hydrophobic character, showing the potential utility of the films in, for example, packaging applications.

## 2 Experimental Section

### 2.1 Materials

Pregelatinized (cold water swellable) native potato starch (Paselli WA4) was kindly supplied by Avebe and dried at 110 °C overnight before use. Oleic acid (90%), carbonyl diimidazole (CDI, ≥ 97%), chloroform-d (CDCl_3_, 99.8%), *N*,*N*′-dicyclohexylcarbodiimide (DCC > 99%), 4-dimethylaminopyridine (DMAP, ≥ 99%), 3-mercaptopropionic acid (MPA, ≥ 99%), polyethylene glycol *M*_n_ = 1000 (PEG_1000_, for synthesis), polyethylene glycol *M*_n_ = 2000 (PEG_2000_, for synthesis), ethylene glycol bis-mercaptoacetate (MA, ≥ 95.0%), 2,2-dimethoxy-2-phenylacetophenone (DMPA, 99%), and methyltetrahydrofuran (≥99%) were all purchased from Sigma–Aldrich. LA (≥99%) was purchased from Myprotein, dl-dithiothreitol (DTT, ≥98%) was purchased from TCI Chemicals, and ethylene glycol (EG, ≥ 99%) was purchased from Emplura. Other common solvents were used with a minimum grade of ≥99.7%. All chemicals were used as purchased, unless stated otherwise.

### 2.2 Methods

#### 2.2.1 Synthesis of SO

Starch, having three hydroxy groups in each repeating unit, was esterified with oleic acid as described in our previous work and the Supporting Information.^3^ The synthesis yielded an SO with a degree of substitution (DS_Oleate_) of 2.2, meaning on average, 2.2 of the hydroxyl group in each repeating anhydroglucose unit is substituted. The corresponding ^1^H NMR spectrum is depicted in Figure S1.

#### 2.2.2 Synthesis of PEG Dilipoic Acid Cross-linker (LA-PEG-LA)

LA-PEG-LA cross-linkers were synthesized by a Steglich esterification, and the synthesis was carried out according to a previously published procedure and is similar for PEG_1000_ and PEG_2000_.^17^ The method with PEG_2000_ is given below as an example. PEG_2000_ (2.00 g, 1 mmol) was dissolved in dichloromethane (DCM, 10 mL), and the solution was purged with nitrogen for 20 min. A second flask was placed on ice under nitrogen after which it was filled with 10 mL of DCM, followed by the addition of LA (2.06 g, 10 mmol, 5 equiv per hydroxy group in PEG), DCC (2.06 g, 10 mmol, 5 equiv per OH), and DMAP (0.21 g, 1.69 mmol, 1 wt % to LA). The flask was kept in the dark to avoid self-polymerization of LA. The LA solution was stirred on ice for 1 h, after which the PEG solution was added. After 1 h, the ice bath was removed and the solution was stirred overnight. A solid side product precipitated from the solution during the reaction and was removed by vacuum filtration. The organic solution was concentrated under vacuum by a rotary evaporator, followed by extraction with a sulfuric acid solution (20 mL, 0.1M) to remove DMAP. The cross-linker was washed in diethyl ether (200 mL) until no unreacted LA was present. The final product was subsequently dried under vacuum.

*LA-PEG*_*2000*_*-LA*: ^1^H NMR (400 MHz, CDCl_3_) δ: 4.22 (4 H, t, −CO–O–C*H*_*2*_−), 3.68 (4 H, t, −CO–O–CH_2_–C*H*_*2*_−), 3.63 (∼174 H, bs, [−C*H*_*2*_–C*H*_*2*_–O−]_1912_), 3.56 (2 H, m, −S–S–C*H–*), 3.12 (4 H, m, −C*H*_*2*_–S–S−), 2.47 (2 H, m, −C*H*_*2*_–CH_2_–S–S−), 2.34 (4 H, t, −S–S–CH–CH_2_–CH_2_–C*H*_*2*_−), 1.90 (2 H, m, −C*H*_*2*_–CH_2_–S–S−), 1.66 (4 H, m, −S–S–CH–C*H*_*2*_−), 1.47 (4 H, m, −S–S-CH–CH_2_–C*H*_*2*_−).

*LA-PEG*_*1000*_*-LA:*^1^H NMR (400 MHz, CDCl_3_) δ: 4.22 (4 H, t, −CO–O–C*H*_*2*_−), 3.68 (4 H, t, −CO–O–CH_2_–C*H*_*2*_−), 3.63 (∼87 H, bs, [−C*H*_*2*_–C*H*_*2*_–O−]_912_), 3.56 (2 H, m, −S–S–C*H*−), 3.12 (4 H, m, −C*H*_*2*_–S–S−), 2.44 (2 H, m, −C*H*_*2*_–CH_2_–S–S−), 2.34 (4 H, t, −S–S-CH–CH_2_–CH_2_–C*H*_*2*_−), 1.90 (2 H, m, −C*H*_*2*_–CH_2_–S–S−), 1.66 (4 H, m, −S–S–CH–C*H*_*2*_−), 1.47 (4 H, m, −S–S-CH–CH_2_–C*H*_*2*_−).

#### 2.2.3 Synthesis of EG Dilipoic Acid Cross-linker (LA-EG-LA)

Initial attempts to synthesize LA-EG-LA afforded a product that could not be isolated; therefore, an equimolar amount of LA (3.32 g, 16.10 mmol) to hydroxy groups (16.10 mmol, 0.50 g of EG) was used instead of the excess as done for the higher molecular weight PEG. The workup only involved vacuum filtration of the precipitated side product, extraction of DMAP with a sulfuric acid solution (0.1M, 20 mL), and removal of the solvent by rotary evaporation, after which the solid product was dried under vacuum at room temperature. ^1^H NMR (400 MHz, CDCl_3_) δ: 4.28 (4 H, S, −C*H*_*2*_–C*H*_*2*_–O−), 3.56 (2 H, m, −S–S–C*H*−), 3.12 (4 H, m, −C*H*_*2*_–S–S−), 2.44 (2 H, m, −C*H*_*2*_–CH_2_–S–S−), 2.34 (4 H, t, −S–S-CH–CH_2_–CH_2_–C*H*_*2*_−), 1.90 (2 H, m, −C*H*_*2*_–CH_2_–S–S−), 1.66 (4 H, m, −S–S–CH–C*H*_*2*_−), 1.47 (4 H, m, −S–S–CH–CH_2_–C*H_2_–*).

#### 2.2.4 Synthesis of the PEG Dimercaptopropionic Acid Cross-linker (MPA-PEG-MPA)

The syntheses of PEG_1000_ and PEG_2000_ were similar, with both being based on a previously published method.^18^ The synthesis of PEG_2000_ is stated below as an example. A round-bottomed flask with 2.00 g of PEG_2000_ (1 mmol) and 30 mL of toluene were equipped with a Dean–Stark trap and a reflux condenser. The system was heated to 120 °C under a nitrogen atmosphere to dry PEG azeotropically, after which it was cooled to 80 °C. MPA (4.25 g, 40 mmol, 20 equiv per OH), DTT (0.077 g, 1.25 mol %), and p-TSA (0.076 g, 1 mol %) were then added, after which the temperature was raised to 115 °C and the mixture allowed to stir overnight. The solvent was removed by rotary evaporation, after which the product was mixed in 10 mL of DCM and then washed with 20 mL of water. The organic layer was precipitated in 200 mL of cold diethyl ether. The product was redissolved in 10 mL of DCM and precipitated in 200 mL of cold diethyl ether two times more. The product was collected and dried under vacuum.

*MPA-PEG*_*2000*_*-MPA:*^1^H NMR (400 MHz, CDCl_3_) δ: 4.27 (4 H, t, HS–CH_2_–CH_2_–CO–O–C*H*_*2*_−), 3.70 (4 H, t, HS–CH_2_–CH_2_–CO–O–CH_2_–C*H*_*2*_−), 3.63 (∼174 H, [−C*H*_*2*_–C*H*_*2*_–O−]_1912_), 2.76 (4 H, m, HS–C*H*_*2*_−), 2.68 (4H, t, HS–CH_2_–C*H*_**2**_−), 1.67 (2 H, t, *H*S−).

*MPA-PEG*_*1000*_*-MPA:*^1^H NMR (400 MHz, CDCl_3_) δ: 4.26 (4 H, t, HS–CH_2_–CH_2_–CO–O–C*H*_*2*_−), 3.70 (4 H, t, HS–CH_2_–CH_2_–CO–O–CH_2_–C*H*_*2*_−), 3.63 (∼87 H, [−C*H*_*2*_–C*H*_*2*_–O−]_912_), 2.76 (4 H, m, HS–C*H*_*2*_−), 2.68 (4 H, t, HS–CH_2_–C*H*_*2*_−), 1.67 (2 H, t, *H*S−).

#### 2.2.5 Film Casting

The films were prepared based on the molar ratio of thiol/sulfur groups to the SO double bond. The following double thiol/sulfur:double bond ratios were used: 1:1, 1:1.5 1:2, and 1:4. The total starch and cross-linker amount was kept at 1.5 g and dissolved in 50 mL of 2-methyltetrahydrofuran. To the solution was added DMPA (4 wt % based on the total film weight), and the solution was degassed by sonication for 30 min. After sonication, the solution was poured into a Teflon tray (10 cm by 10 cm) and then left to evaporate in the dark, resulting in the formation of the corresponding films. A small sample of each film was taken and stored in the dark for further analysis.

#### 2.2.6 UV Curing

After the films were fully dried they were irradiated under UV light for 1 h (365 nm) to cross-link the material.

### 2.3 Characterization

#### 2.3.1 ^1^H NMR Spectroscopy

A Varian 400 MHz instrument was used to record the ^1^H NMR spectra. Chloroform-d_6_ (7.14 ppm) was used as the solvent and was set as the reference peak. The degree of substitution (DS_Oleate_) of the SO was calculated via eq 1. The terminal −CH_3_ group of the oleate chain was used to normalize the integration and was set to 3. *X* in eq 1 refers to the integration in the region between 4.7 and 6.0 ppm, representing the protons in the starch anhydroglucose ring and the protons of the double bond of the fatty acid. When these values are known, the DS_Oleate_ can be calculated.^2^
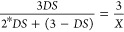
1

The extent of esterification of PEG_1000_ and PEG_2000_ with MPA or LA was calculated with eq 2 and eq 3. *M*_n,PEG_ is the molecular weight of PEG used. The value of *M*_n,PEG_ was subtracted by the molecular weight of the terminal EG units (88 g·mol^–1^) of PEG, divided by the molecular weight of the PEG repeating units (44 g·mol^–1^), and multiplied by 4 (the number of protons in one repeating unit) to obtain *A*, which is the number of protons that refer to the signal at 3.63 ppm.
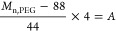
2

When the peak at 3.63 ppm was set to the corresponding integration (*A*), a value for integration (*B*) follows from the peak that corresponds to the terminal PEG repeating units that were esterified with MPA or LA at 4.26 ppm. In the reaction between EG and LA, eq 2 is not needed.
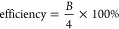
3

#### 2.3.2 Fourier Transform Infrared Spectroscopy (FTIR)

FTIR spectra were measured using the attenuated total reflectance (ATR) mode on a vertex 70 Bruker spectrometer, at a resolution of 16 cm^–1^, and with a scan time of 32 s for both the sample and background scans. The background was corrected and the spectra normalized to the peak at 1030 cm^–1^ with OPUS software.

#### 2.3.3 Rheometry

Frequency sweep measurements were performed on an Anton Paar Physica MCR 302e rheometer using plate–plate geometry. The measurements were performed in the elastic region, which corresponds to a strain of 0.2%. The films were measured from 100 to 1 Hz at 60 °C.

#### 2.3.4 Differential Scanning Calorimetry (DSC)

The thermal transitions were determined on a Q1000 instrument from TA Instruments. The test was performed from −80 to 180 °C using a modulated program in which the temperature increased by 2 °C·min^–1^. The data were analyzed with TRIOS software.

#### 2.3.5 Thermogravimetric Analysis (TGA)

Degradation temperatures (*T*_d_’s) were established by TGA carried out on a TA Instruments 5500 instrument under a nitrogen atmosphere. The measurement ran from 25 to 700 °C with a heating rate of 10 °C·min^–1^. Similar to the DSC measurements, the data were analyzed with the TRIOS software.

#### 2.3.6 X-ray Diffraction

X-ray diffraction patterns of the films were obtained on a Bruker D8 ADVANCE apparatus. The films were measured between 2θ angles of 4 and 50° using a Cu Kα radiation source with λ = 0.1542 nm.

#### 2.3.7 Contact Angle

The hydrophobicity of the films was determined by contact angle analysis. The contact angle was determined by adding a deionized water drop (2 μL) to the film surface with a VCA-2500XE (AST) system. Every film was measured in triplicate, and averages with standardization were derived from these.

#### 2.3.8 Tensile Testing

Dog bone-shaped halters were cut from the films, and their mechanical properties were tested on an Instron tensile tester 5565, which had a load cell of 100 N. The films were elongated at 2 mm·s^–1^. All different sample types were measured five times, and the results were reported as averages with standard deviations.

#### 2.3.9 Statistical Analyses

Where applicable, the data were analyzed using one-way analysis of variance (ANOVA) with Tukey’s test in SPSS statistics software (version 26, IBM, New York, New York, USA). Data are considered to be significantly different when *P* < 0.05.

## 3 Results and Discussion

### 3.1 Cross-linker Characterization

**Figure 1:**
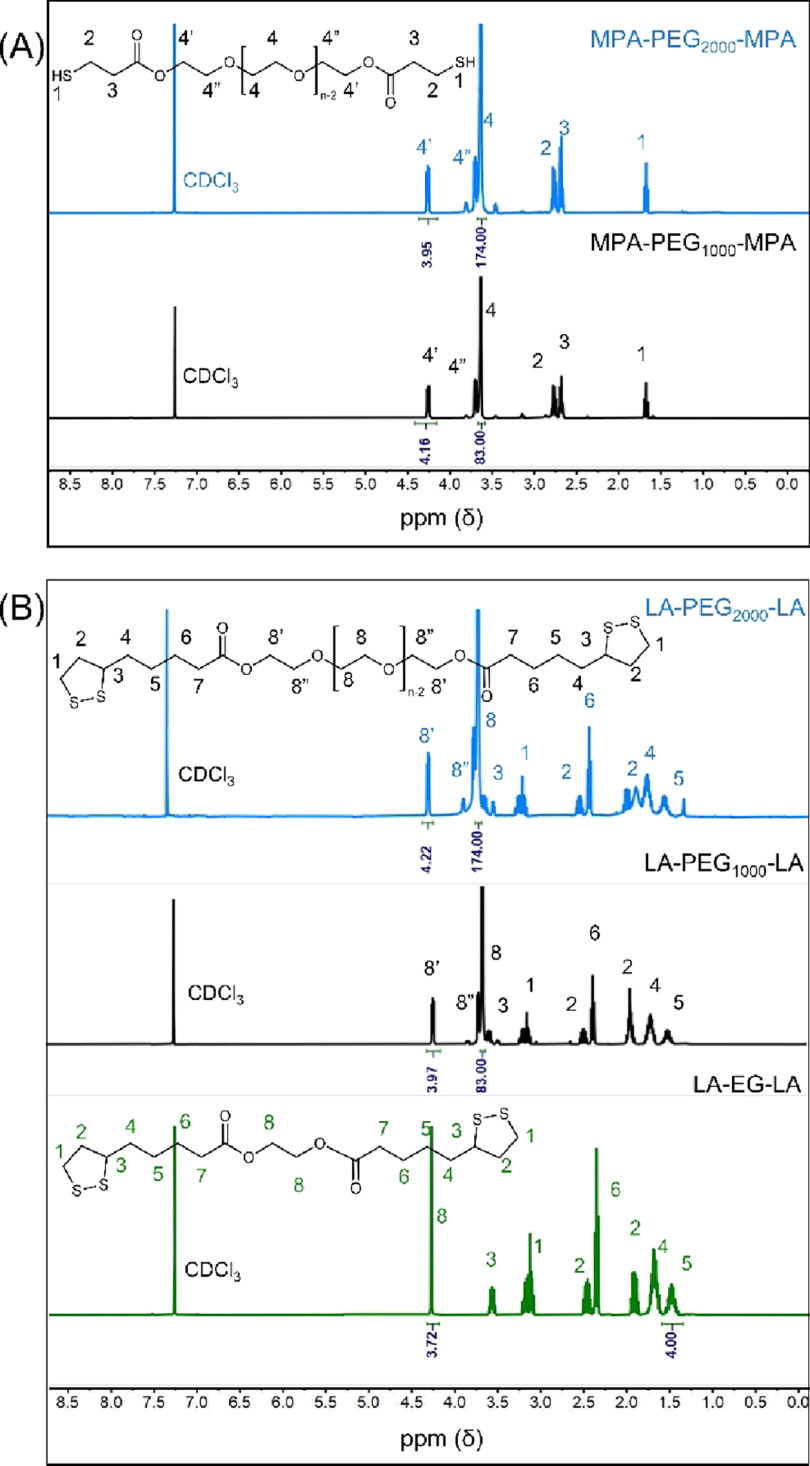
^1^H NMR spectra of the cross-linkers (A) MPA-PEG_2000_-MPA (top) and MPA-PEG_1000_-MPA (bottom) and (B) LA-PEG_2000_-LA (top), LA-PEG_1000_-LA (middle), and LA-EG-LA (bottom).

For the full characterization of SO, we refer to our earlier work, as this study focuses on the synthesis and the use of the cross-linkers.^3^ However, the ^1^H NMR spectrum of SO can be found in the Supporting Information (Figure S1). The cross-linkers were synthesized by esterifying PEG_1000_, PEG_2000_, or EG with M(PA) or LA via a simple Fisher or Steglich esterification. LA could not be added by a Fisher esterification as self-polymerization occurs above 60 °C.^19^ All synthesized cross-linkers were analyzed by ^1^H NMR, and the spectra are shown in Figure 1.

Figure 1a shows the spectra of MPA-PEG_1000_-MPA and MPA-PEG_2000_-MPA. Esterification was confirmed by the signal at 4.21 ppm, corresponding to the protons of the terminal repeating PEG units. PEG_1000_ and PEG_2000_ were esterified with MPA for 100 and 98%, respectively. EG was not esterified with MPA since a similar molecule, ethylene glycol bis-mercaptoacetate (MA-EG-MA), is commercially available.

The products obtained after esterification of EG, PEG_1000_, and PEG_2000_ are shown in Figure 1b. All peaks in Figure 1b were successfully assigned, and similar to the esterification of MPA, the signal at 4.21 is indicative of esterification. Based on the integration of the PEG backbone peak and the peak at 4.21 ppm, 99 and 100% of the hydroxyl groups of PEG were esterified with LA for PEG_1000_ and PEG_2000_, respectively.

The reaction between LA and EG reached a 100% degree of esterification. Similar to the esterification of PEG, a signal at 4.28 ppm is present, which indicates esterification. Contrary to PEG, EG does not contain any repeating units; hence, the signal at 3.63 ppm is absent, while for the cross-linked based on PEG_1000_ and PEG_2000_, this peak remained present.

### 3.2 Film Characterization

The casting of SO with the cross-linkers resulted in semitransparent films. The films made with LA-based cross-linkers were slightly yellow, due to the presence of LA. Films containing the MPA-PEG_2000_-MPA cross-linker and having thiol:double bond ratios of 1:1, 1:1.5, and 1:2 were observed to be flakey due to the high PEG_2000_ content. Images of all prepared films can be found in the Supporting Information (Figure S2).

**Figure 2:**
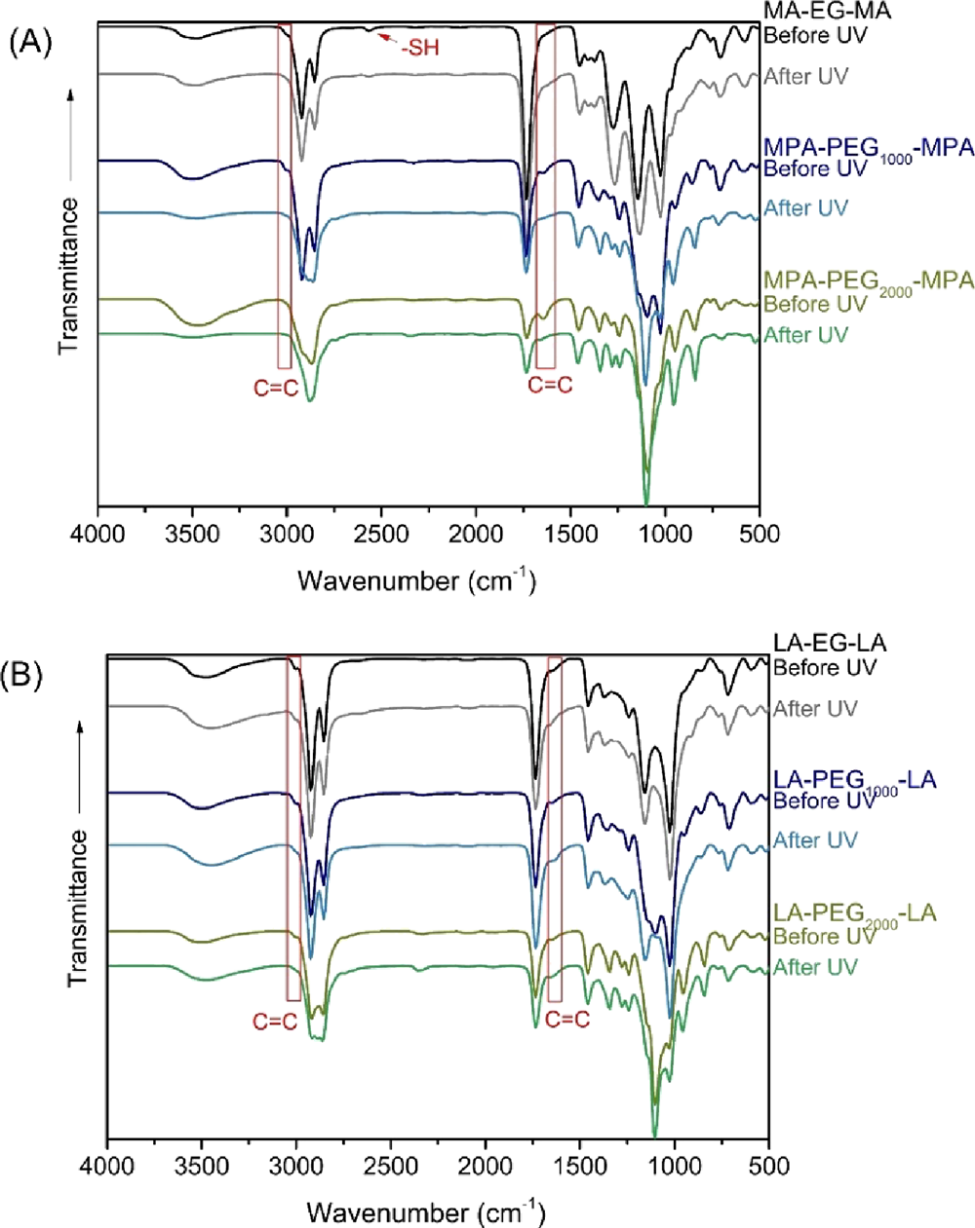
FTIR spectra of SO films with (A) M(P)A-based cross-linkers and (B) LA-based cross-linkers. The spectra were taken from films that contain a 1:1 ratio of thiol/sulfur:double bonds. Both show spectra before and after UV irradiation.

Cross-linking was verified by the presence of the SO double bond in the FTIR spectra. In the case of M(P)A-based cross-linkers, this was also done by the presence of the thiol groups. Figure 2a shows the FTIR spectra of films with M(P)A-based cross-linkers before and after UV irradiation. For uncross-linked films, the double bond was present at 3005 and 1647 cm^–1^ while the signal corresponding to the thiol appears at 2571 cm^–1^. Figure 2a shows that the films with MPA-EG-MPA and MPA-PEG_1000_-MPA have a band at 3005 cm^–1^ that disappears after UV irradiation, indicating cross-linking of the double bond, which is in agreement with the literature.^18,20^ In films with the MPA-PEG_2000_-MPA cross-linker, no peak was visible, even before UV irradiation. PEG has a signal at a similar wavenumber (3005 cm^–1^) and overlaps with the double bond signal. Due to the high weight fraction of PEG, the double bond signal is not visible.^21^

The other band corresponding to the oleate double bond at 1647 cm^–1^ was visible for films with MPA-PEG_2000_-MPA cross-linkers before UV irradiation. This peak fully disappeared after UV irradiation, consistent with cross-linking having occurred.^15,22^

In addition to the presence of the double bond, cross-linking can be verified by the presence of thiols at 2500 cm^–1^. As the weight fraction of EG in MA-EG-MA-based films is much lower than in films with PEG-based cross-linkers, the free thiol moiety is only visible in this FTIR spectrum before UV irradiation. This signal disappears after UV irradiation, again consistent with cross-linking having occurred.^15,22^

During cross-linking, C–C, C–O–C, C–S, and S–S bonds will be formed. The S–S bond is known to undergo side reactions in which the bond is again broken followed by a reaction with a hydroxy group. The radical process of cross-linking and the formation of side products are schematically depicted in Scheme S1 in the Supporting Information. Since starch was not fully substituted, the starch ester still contained hydroxy groups that can participate in this side reaction, lowering the intensity of the band at 3500 cm^–1^.^11,23^ Although this is considered a side reaction, it still contributes to the extent of cross-linking the material.

Figure 2b shows the FTIR spectra of films containing the LA-based cross-linker before and after UV irradiation. All spectra show a signal before UV irradiation at 3005 cm^–1^, corresponding to the SO double bond. The LA-based cross-linker contains four sulfur atoms per cross-linker that can participate in cross-linking, whereas the M(P)A-based cross-linker only has two. Hence, the weight fraction of the LA-based cross-linker was lower, meaning that the number of unsaturated bonds in the film before curing was higher, as was the signal intensity. After UV irradiation, this band disappears in all LA-based films. In addition to the band at 3005 cm^–1^, a second signal from the double bond appears at 1647 cm^–1^. After UV irradiation, this band merges with the band at 1735 cm^–1^, which is typical for cross-linking.^22^ Contrary to the M(P)A-based cross-linker, there are no free thiols, meaning that cross-linking can only be verified by the absence of the unsaturated double bond.^15^ Although LA is known to undergo a similar side reaction (Scheme S1) involving the disulfide ring, no change was visible in the hydroxyl band before and after irradiation with UV light.^23^ The spectra of the SO films with lower ratios of cross-linkers can be found in the Supporting Information (Figure S3).

**Figure 3:**
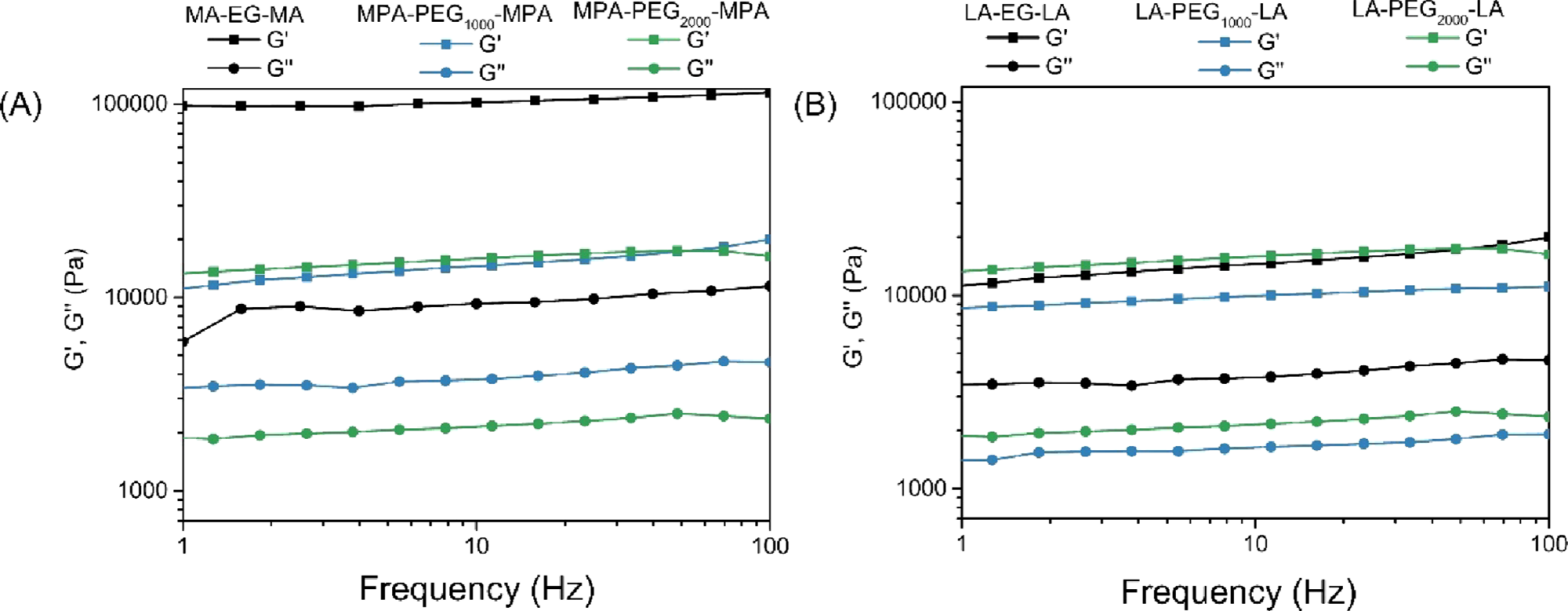
Storage (*G*′) and loss (*G*″) modulus of SO films cross-linked with (A) M(P)A-based cross-linkers and (B) LA-based cross-linkers.

In addition to FTIR, films with a 1:1 ratio of thiol/sulfur:double bonds were analyzed by dynamic oscillatory rheometry to confirm cross-linking. The results of the measurements shown in Figure 3 reveal that the storage modulus (*G*′) is higher than the loss modulus (*G*″) for all films, indicating a solid material. The storage moduli are nearly constant over the measured frequency range of 1 to 100 Hz, indicating a cross-linked material.

Both figures also show that loss moduli are higher for longer chain lengths, meaning that the materials become softer with increasing molecular weight of PEG. This is as expected since the melting point (*T*_m_) of the cross-linker (Figure S5) increases with increasing molecular weight.

### 3.3 Thermal Properties

**Figure 4:**
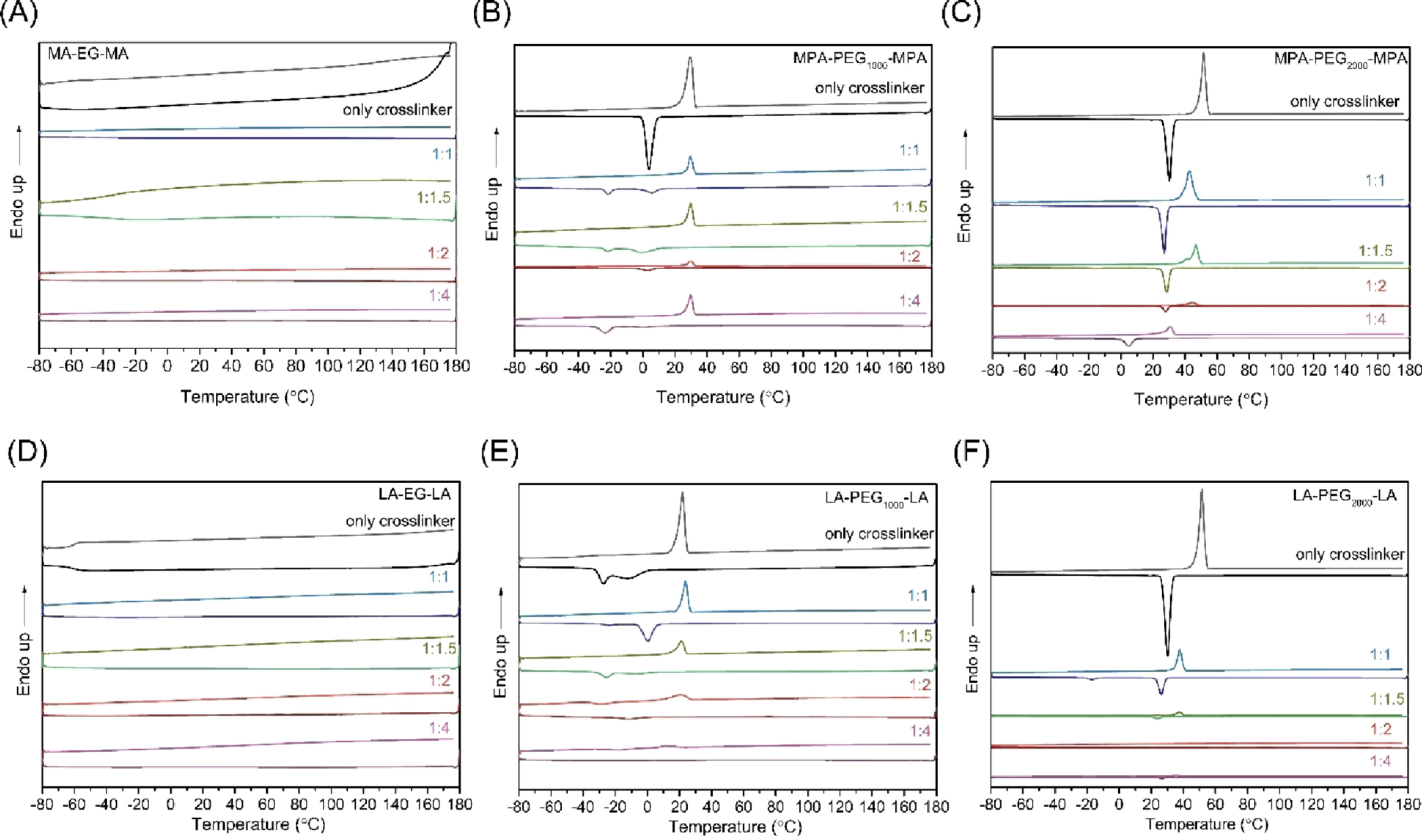
DSC thermograms showing the first cooling cycle and second heating cycle of the SO films with different cross-linker functional ends, lengths, and ratios to double bonds. Showing SO with (A) MA-EG-MA, (B) MPA-PEG_1000_-MPA, (C) MPA-PEG_2000_-MPA, (D) LA-EG-LA, (E) LA-PEG_1000_-LA, and (F) LA-PEG_2000_-LA.

By incorporating PEG-based cross-linkers with varying *T*_m_, the thermal properties of the SO films cross-linked with those cross-linkers are expected to change. These properties were measured by DSC for all different cross-linker lengths and different ratios of cross-linker to SO, and the resulting spectra are shown in Figure 4.

Un-cross-linked SO has a *T*_g_ visible at 93 °C before the addition of the cross-linker, which has been shown to disappear upon direct cross-linking of the oleate chains.^4^ Similar behavior is expected when cross-linking SO with PEG-based cross-linkers, although new thermal transitions are expected to arise with the addition of the cross-linker as well.

The effect of cross-linking of SO with MA-EG-MA on the thermal properties is shown in Figure 4a. The spectra do not show any *T*_g_ or *T*_m_, regardless of cross-linker content. As expected, the *T*_g_ observed for uncross-linked SO is no longer present.

As a control, cross-linked films consisting solely of cured cross-linkers were measured and are shown in the same figure. In addition, the *T*_m_ of the cross-linkers without any UV irradiation was determined; the results can be found in the Supporting Information (Figure S5). Similar to the SO films with the MA-EG-MA cross-linker, no *T*_g_ or *T*_m_ was observed in both cases.

For SO films cured with MPA-PEG_1000_-MPA, a *T*_m_ is observed at 30 °C. In the cooling cycle, two crystallization peaks are visible at −22 and 2 °C and are present for every ratio of the double bond to thiol investigated. Cross-linked MPA-PEG_1000_-MPA without SO showed a similar *T*_m_ at 30 °C and a crystallization peak at 4 °C. Upon mixing of the cross-linker with the SO, a second crystallization peak again arises at −22 °C and the intensity of this peak increases with increasing cross-linker content. Since this peak is absent in films comprising exclusively SO or cross-linker, it originates from the crystallization between SO and the PEG-based cross-linker.

Further increasing the PEG molecular weight resulted in an increase in *T*_m_ and *T*_c_ for the cured film consisting solely of MPA-PEG_2000_-MPA, which were 52 and 30 °C, respectively. When this cross-linker was mixed with SO in a 1:1 ratio of thiol:double bond and then cured, *T*_m_ and *T*_c_ both decreased to 43 and 27 °C, respectively, and kept decreasing to *T*_m_ = 30 °C and *T*_c_ = 4 °C for a 1:4 ratio of thiol:double bond.

SO films with the LA-EG-LA cross-linker at a 1:1 ratio of sulfur:double bond showed a small step in the curve, which indicates a *T*_g_ at −38 °C. This *T*_g_ arises from the LA-EG-LA cross-linked with itself. A cross-linked film prepared solely from LA-EG-LA shows a *T*_g_ at −60 °C (Figure 4d), which arises due to the disulfide polymer backbone of polylipoic acid.^24,25^ When the cross-linker content is decreased, the *T*_g_ is no longer present. Since less cross-linker was present, the ability of the disulfide network to cross-link with itself instead of to the double bond of SO was lower. Even in films with low cross-linker content, no *T*_g_ was present. This probably means that the SO double bonds have reacted with themselves, since the number of double bonds is higher than the number of thiol groups.

Figure 4e shows the spectra of cross-linked LA-PEG_1000_-LA and of films consisting of SO with different LA-PEG_1000_-LA ratios. For the films made from only the cross-linker, a *T*_g_ is observed at −37 °C, corresponding to the polymerized LA backbone. This heating cycle also shows a *T*_m_ at 22 °C, corresponding to the melting of the crystalline phase of PEG. This crystalline phase is responsible for both crystallization peaks in the cooling cycle, namely, a broad signal peak at −11 °C followed by a sharper signal at −28 °C. When this cross-linker was cured with SO in a 1:1 ratio of thiol:double bond, the *T*_g_ decreased to −47 °C; however, the melting peak remained similar at 24 °C, comparable to the *T*_m_ of LA-EG-LA. The cooling cycle again shows two crystallization peaks in which the first was sharper and now present at 0 °C and the second at −24 °C. With lower cross-linker content, *T*_m_ decreases slightly from 21 to 15 °C and then 10 °C while *T*_c_ remains relatively unchanged. The decrease in the *T*_m_ with decreased PEG content is consistent with other research.^26^ Both crystallization peaks decreased in size, transitioned into one broad peak, and were no longer present at the lowest cross-linker concentration. Cross-linking of SO to PEG prohibits PEG crystallization, leading to a decrease in *T*_c_ as well as its magnitude.^26^ However, no crystallization peak was present in the cooling cycle while a cold crystallization signal was present in the heating cycle at −18 °C.

Cross-linked LA-PEG_2000_-LA has a *T*_m_ at 38 °C and a *T*_c_ at 25 °C. Upon mixing and curing with SO in a 1:1 ratio of sulfur:double bond, similar values for *T*_m_ and *T*_c_ were found compared to the cured film without SO (Figure 4F). However, a second *T*_c_ was visible at −18 °C, which was moreover most pronounced at the highest concentration of the cross-linker, as expected. Cross-linkers with similar cross-linker sizes always show a lower *T*_m_ for LA-based cross-linkers than for M(P)A-based cross-linkers, following the same trend in *T*_m_ as the uncured cross-linker, as shown in the Supporting Information (Figure S5).

**Figure 5:**
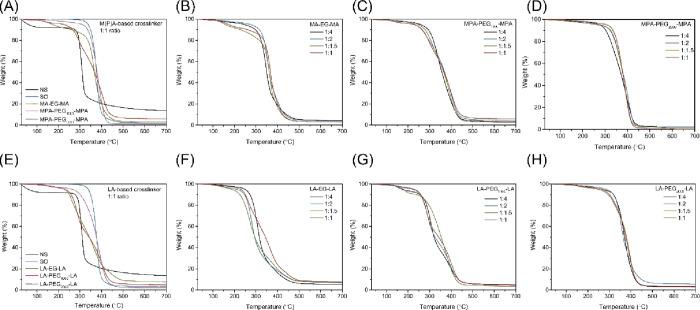
Thermograms of SO films with different cross-linker functional ends, lengths, and ratios to double bonds. Showing the *T*_d_ of (A) native starch (NS), SO and SO with MPA-based cross-linker, (B) SO with MA-EG-MA, (C) SO with MPA-PEG_1000_-MPA, (D) SO with MPA-PEG_2000_-MPA, (E) NS) SO and SO with the LA-based cross-linker, (F) SO with LA-EG-LA, (G) SO with LA-PEG_1000_-LA, and (H) SO with LA-PEG_2000_-LA.

In addition to the *T*_g_, the effect of cross-linking with the (P)EG-based cross-linker on the degradation temperature (*T*_d_) was studied by TGA (Figure 5).

Figure 5a shows that curing SO with the M(P)A-based cross-linker lowers the onset in *T*_d_ compared to pure SO films without a cross-linker. Films with lower molecular weight cross-linkers processed an earlier onset in *T*_d_, consistent with the *T*_d_ of pure PEG.^27^ During cross-linking, C–S and S–S bonds are formed, which are less heat resistant than C–C bonds, causing the onset of the *T*_d_ of the cross-linked films to decrease.^28^ Although the onset in *T*_d_ might decrease, the temperature at which weight loss is maximum is similar to that of SO irrespective of the cross-linker size. Figure 5b–d shows that the cross-linker ratio to double bond had little effect on *T*_d_.

Figure 5a,e shows that native starch has the highest char yield compared to all modified starches. Native starch has a char yield of 13.7% due to its strong intermolecular hydrogen bonding.^29^ In modified starch, those hydroxy groups are substituted and can therefore no longer participate in cross-linking, resulting in uncross-linked SO having a char yield of 2.0%. For some cross-linked films, the char yield is slightly higher as is most obvious in Figure 5e. A slight increase (up to 7%) is mainly observed for the films with higher cross-linker content, which will result in a higher cross-linker density which typically increases the char yield.^4^

SO cured with LA-based cross-linkers possess a *T*_d_ of approximately 50 °C lower than films with M(P)A-based cross-linkers, consistent with the literature.^15^Figure 5e again shows that *T*_d_ increases with increasing cross-linker length. Within the different ratios of thiol/sulfur:double bonds, similar degradation curves were obtained. However, all curves show a broader degradation range than SO. In the LA-based cross-linker from EG and PEG_1000_, a two-step degradation was visible with onset temperatures of 220 and 250 °C_,_ respectively. The second degradation-stage ranged from 355 to 450 °C and arises from the SO part of the films.

### 3.4 Structural Characterization

**Figure 6:**
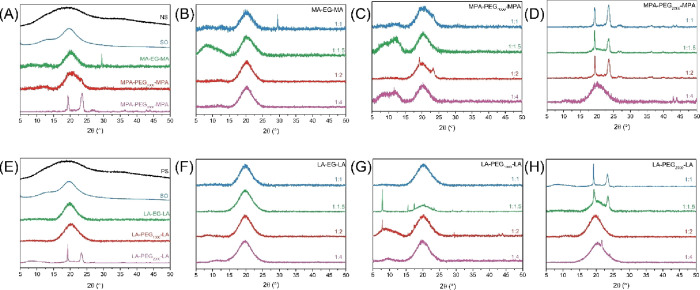
XRD spectra of SO films with different cross-linker functional end groups, lengths, and ratios to the double bond. The figures represent (A) native starch (NS), SO and SO with M(P)A-based cross-linker at the 1:1 ratio, (B) SO with MA-EG-MA, (C) SO with MPA-PEG_1000_-MPA, (D) SO with MPA-PEG_2000_-MPA, (E) native starch (PS), SO and SO LA-based cross-linker at the 1:1 ratio, (G) SO with LA-PEG_1000_-LA, and (H) SO with LA-PEG_2000_-LA.

Curing of SO with (P)EG-based cross-linkers is expected to increase the crystallinity of the materials. Therefore, the materials were studied by XRD and the results are depicted in Figure 6. Cold water swellable potato starch is completely amorphous, and esterification with oleic acid resulted in a material with a higher crystalline order (Figure 6a).^2^ Upon cross-linking of SO with MPA-PEG_2000_-MPA, the crystallinity increased further, as revealed by two sharp peaks which are typical for the crystal structure of PEG.^13^ Those peaks are absent when SO is cross-linked with lower molecular weight M(P)A-based cross-linkers. Figure 6b shows that the MA-EG-MA cross-linker had little effect on film crystallinity regardless of the cross-linker content. This is in agreement with the DSC results, in which no *T*_m_ was observed.

Figure 6c represents films with the MPA-PEG_1000_-MPA cross-linker at different ratios, and they show a similar degree of crystallinity as the MA-EG-MA cross-linker, although DSC analysis does show a *T*_m_. The PEG peak intensity might overlap with the SO peaks and, moreover, might not be intense enough to be visible.

The diffraction patterns in Figure 6d indicate that the crystallinity induced by MPA-PEG_2000_-MPA increased with increasing cross-linker content, as the weight fraction of PEG increased, which is in line with the DSC results where the area of the *T*_m_ was increased as well. This means that the major contribution to crystallinity is from the PEG unit and not SO.

The films with LA-based cross-linkers showed a similar diffraction pattern as M(P)A-based films. No change in crystallinity was observed when curing with LA-EG-LA, which is in agreement with the DSC results in which the *T*_m_ is absent. Similar to SO films with the MPA-PEG_1000_-MPA cross-linker, films with the LA-PEG_1000_-LA have a diffraction pattern similar to that of pure SO. The *T*_m_ of these films is around room temperature, which further explains the absence of the PEG crystalline domains. Moreover, the ratios 1:1.5 and 1:2 of the sulfur:double bond show peaks at 8, 16, and 17° that are typical for LA.^30^

Finally, Figure 6h shows that the peaks of the PEG crystals are less pronounced than in the MPA-based cross-linker with comparable molecular weight. The LA-based cross-linker has four cross-linking points, lowering the weight fraction of the cross-linker in the films. The peak at 23° has an increased intensity compared to the peak at 19°. This is an indication of a change in the orientation of the orthorhombic crystal structure.^31^ Besides peaks corresponding to the cross-linker, Figure 6b,c,g shows a peak at 7°, which arises due to stacking of the oleate chains.^2^

### 3.5 Surface Wettability

**Figure 7:**
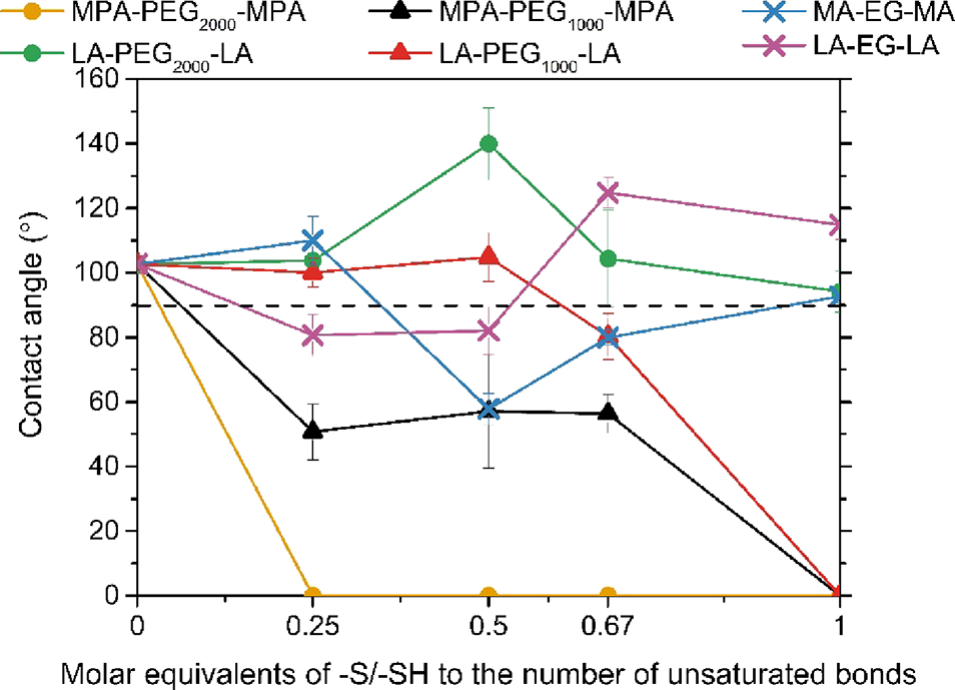
Contact angles of the SO films with different cross-linkers.

(P)EG is hydrophilic and therefore might affect the hydrophobicity of the films; thus, the surface wetting of the films was tested (Figure 7). Before cross-linker addition, SO has a contact angle of 102°.^3^Figure 7 shows that the main difference in contact angle was based on whether LA or MPA was used as a cross-linking unit. The M(P)A-based cross-linker shows a decrease in contact angle with increasing spacer length, with complete wetting for all films containing the MPA-PEG_2000_-MPA cross-linker, irrespective of the amount of cross-linker present. In contrast, films with LA-PEG_2000_-LA were hydrophobic even at a 1:1 ratio of sulfur:double bond.

However, the LA-based cross-linker contains double the amount of functional groups than the M(P)A-based cross-linkers, resulting in less cross-linker being needed to obtain the same ratio of thiol/sulfur:double bonds. This means that the films with LA-based cross-linkers contain less PEG, which was responsible for the decrease in hydrophobicity due to the ether bond and explains why those films had a higher contact angle than the films based on M(P)A-based cross-linkers.^13^ In addition, the LA-based cross-linkers have hydrophobic ends while M(P)A-based cross-linkers are hydrophilic end groups. This might result in the LA end groups forming a hydrophobic core around the hydrophilic PEG units, therefore shielding the effect of the hydrophilic ether groups.^32−34^ This agrees with the XRD data that reveal a difference in orientation of the crystal structure for LA- and M(P)A- based films.

In addition to the effect on the cross-linker end group, the influence on the cross-linking content was examined. When employing MPA-based cross-linkers, the contact angle decreased with increasing cross-linker content, with one exception being the film with the MA-EG-MA cross-linker at 0.5 mol equiv of thiols to the double bonds. However, this particular film has a substantial large standard deviation, placing its value within a range similar to that seen with a 0.67 equivalence. This decrease in contact angle was expected with increasing cross-linker content as a higher cross-linker content introduces more hydrophilic ether groups into the system. In contrast, the films from LA-based cross-linkers appear to be less influenced by the variation in cross-linker content. As previously mentioned, the LA-based cross-linker might create a hydrophobic core that shields the ether groups. As the cross-linking content increases, the PEG content follows. The PEG content is shielded and, thus, has little effect on the content angle. Notably, the contact angle for films with the LA-PEG_2000_-LA cross-linker at 0.5 equiv is higher compared to sulfur to double bond ratios. The XRD results for this specific film show the absence of sharp peaks corresponding to the stacking of the PEG chains, while at other ratios, those peaks are more pronounced. This is indicative that at this particular ratio, the PEG units are less stacked; instead, the cross-linker is probably more efficient in forming this hydrophobic core and increasing the contact angle, while the opposite might be happening in the film with an LA-PEG_1000_-LA cross-linker at a 1:1 equivalence. No correlation between the cross-linker length and the contact angle was found for the LA-based cross-linkers, as those might be both influenced by the ability of shielding by the hydrophobic end groups and the extent to which stacking of PEG units takes place.

### 3.6 Mechanical Properties

**Figure 8:**
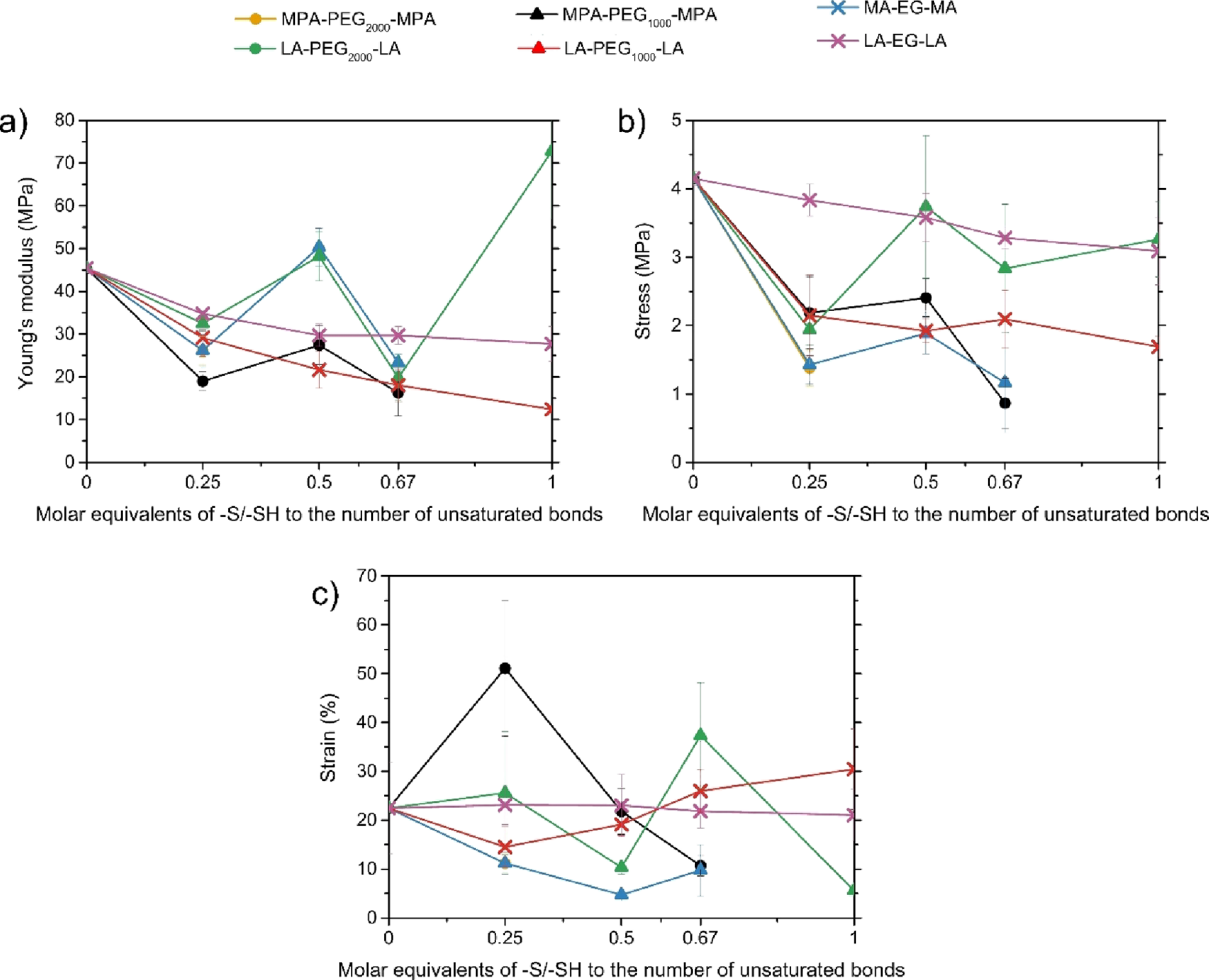
Mechanical properties of the SO films with the different cross-linkers in which (A) shows the Young’s modulus, (B) the stress at break, and (C) the maximum strain.

Last, the film mechanical properties were evaluated and the results are depicted in Figure 8.

The mechanical properties were tested by a tensile tester, and the results are illustrated in Figure 8. Additionally, a stress–strain curve of each film that represents the average of that film is stated in the Supporting Information (Figure S6).

Film incorporating MPA-PEG_2000_-MPA cross-linkers exhibits poor film formation, limiting mechanical analysis. For this cross-linker, only films with a molar equivalence of 0.25 thiol to the unsaturated bond, corresponding to a 1:4 ratio of the thiol:double bond ratio, were suitable for testing. The result revealed a lower maximum strength and strain compared to un-cross-linked SO. This can be attributed to the cross-linked films possessing a higher order of crystallinity induced by PEG, making them more brittle.

M(P)A-based films were more susceptible to the crystallinity induced by PEG than their LA counterparts. This difference stems from M(P)A-based cross-linkers having two functional groups available for cross-linking, while LA-based cross-linkers possess four functional groups. A similar amount of functional groups results in a higher PEG content in M(P)A-based films, making those more susceptible to the crystallinity induced by PEG, resulting in a greater reduction in strain (Figure S6). This effect was most obvious at a 1:1 equiv ratio of functional groups to the double bond for the M(P)A-based films that were too brittle for testing (Figure 8b).

Irrespective of the type of cross-linker used, the maximum strength decreased with increasing the equivalent of cross-linker to the unsaturated bond, which is in agreement with literature.^13,18^ The maximum strength decreased with increasing cross-linker content because PEG acts as a soft spacer, limiting the number of physical intermolecular cross-links between the SO molecules, thereby causing an increase in flexibility and maximum strain of the films.^13^ The film made using the LA-PEG_2000_-LA cross-linker at 0.5 mol equiv of sulfur functional group to the double bonds is an exception. This value, however, does have a large standard deviation, and similar to the contact angle measurements, it is considered an outlier. As previously discussed, in this particular film, it is expected that the cross-linker might not stack but instead forms a cluster, having an opposite effect on the strain. The maximum strain did increase at 0.25 equiv of cross-linker to the unsaturated bond compared to uncross-linked SO, but at high cross-linker content, the maximum strain decreased again due to the crystallization of the PEG segments, as was strongly the case for the films with MPA-PEG_1000_-MPA. In two specific films is the stress considerably larger than in all other films synthesized. This is the case for films with LA-PEG_2000_-LA at 0.67 equivalence, and MPA-PEG_1000_-MPA at 0.25 equivalence. It is worth noting, however, that both of these specific films have considerably large standard deviations, raising questions on the accuracy of this value The elongation at break of the films in this research falls within the range of current plastic films made from LDPE or PP, which have typically an elongation at break of 10 and 70%, respectively. Unfortunately, the yield stress still requires improvement to reach similar values for the tensile stress to be comparable to the current plastics, both of which are around 30 MPa.^35,36^

## 4 Conclusions

PEG_2000_, PEG_1000_, and EG were esterified with either LA or M(P)A. Each cross-linker was mixed with SO at different ratios of thiol/sulfur:double bond and cross-linked by UV irradiation, resulting in the formation of fully biobased films in most cases. Successful cross-linking by UV irradiation was confirmed by FTIR analysis and dynamic oscillatory rheometry. DSC analysis showed that the films with an EG-based cross-linker did not contain any *T*_m_ while *T*_g_ was only visible in LA-based cross-linkers. In contrast, all cross-linkers based on PEG_1000_ and PEG_2000_ showed a *T*_m_ from the crystalline region of PEG, which was confirmed by XRD analysis. TGA revealed that as the cross-linker length increased, the films had a higher *T*_d_. Similarly, the contact angle of the M(P)A-based films decreased with increasing cross-linker length and content while the contact angle for LA-base films was unaffected regardless of the cross-linker content or length. Tensile test results showed that the maximum strength decreased for all films. The elongation at break increased for the films with the MPA-PEG_1000_-MPA cross-linker but was restricted by its crystallinity. LA-based cross-linkers did show an increase in elongation as well and were less affected by the crystallinity.

## Supporting Information Available

The experimental procedure for the synthesis of SO and the corresponding ^1^H NMR spectrum; pictures that show the difference in the physical appearance of the films; the radical process of cross-linking; FTIR spectra of all films discussed before and after UV irradiation; DSC spectra that show the melting points of the different crosslinkers used in this study; and stress strain cure of films with MPA-based crosslinkers and films with LA-basedcrosslinkers (PDF)
