# Supplementary material for: Thiol–Ene Click Cross-linking of Starch Oleate Films for Enhanced Properties
Source: Biomacromolecules. 2023 Nov 7;24(12):5578–88. doi: 10.1021/acs.biomac.3c00507 (PMC10716852; doi:10.1021/acs.biomac.3c00507)
Supplement: Supplementary file 1 — bm3c00507_si_001.pdf [file bm3c00507_si_001.pdf]

Supporting Information

Thiol-ene Click Crosslinking of Starch Oleate Films for Enhanced Properties

Laura Boetje[a], Xiaohong Lan[a], Jur van Dijken[a], Gerbrich Kaastra[b],

Michael Polhuis[c], and Katja Loos[a]*

A round-bottomed flask was placed in an 80 C °oil bath and to this flask, dried potato starch (1 gram) was added and dissolved in DMSO. In a second round-bottomed flask was oleic acid (1.6 eq per OH) dissolved in DMSO. Oleic acid was activated for 30 minutes by 1,1'-carbonyldiimidazole (1 eq. per carboxylic acid) at 60 °C. After those 30 minutes of activation, the oleic acid solution was added to the starch solution and kept stirring at 80 °C overnight resulting in the formation of a gel. After removal of DMSO, the gel was dissolved in $CHCl_3$, and precipitated in ethanol to afford a white powder. The process of redissolving and precipitation was done twice more to remove the remaining DMSO and starting material. The white powder was collected and dried in a 40 °C

vacuum oven.

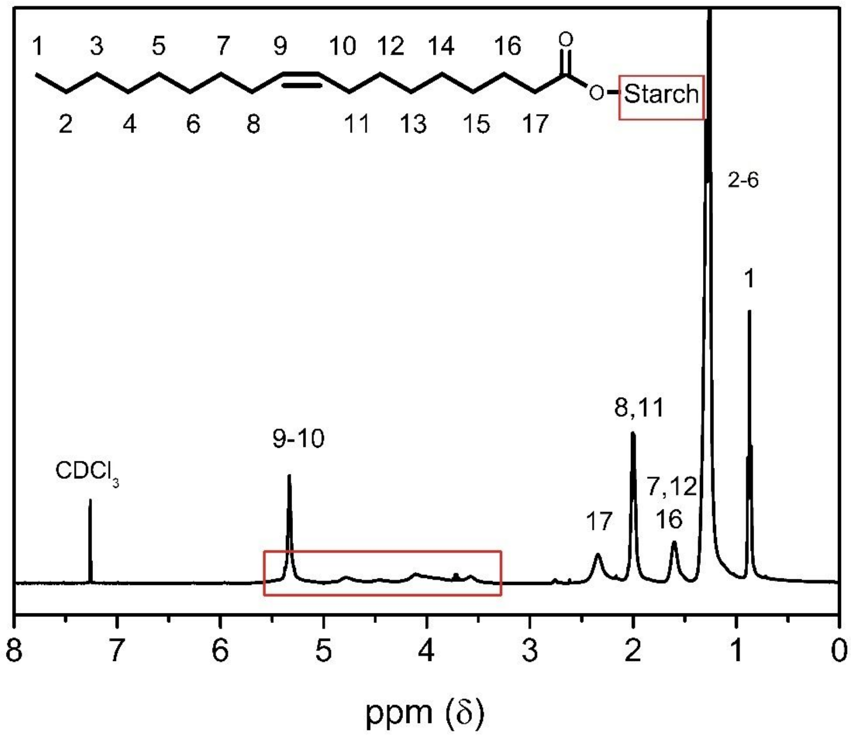

Figure S1. ^{1}HNMR spectrum of starch oleate

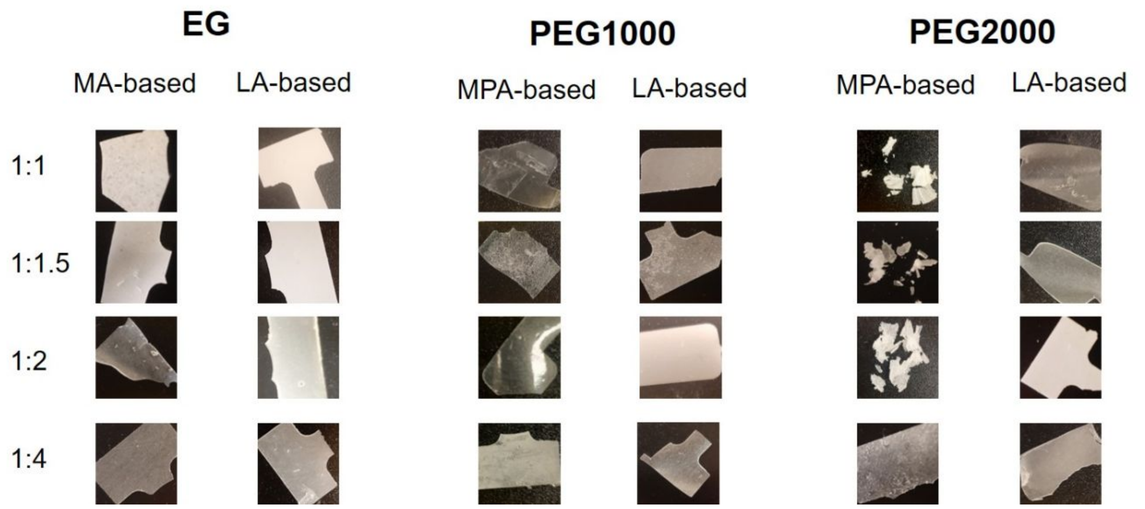

Figure S2. Physical appearance of the SO films with all different crosslinkers used in this study after UV irradiation

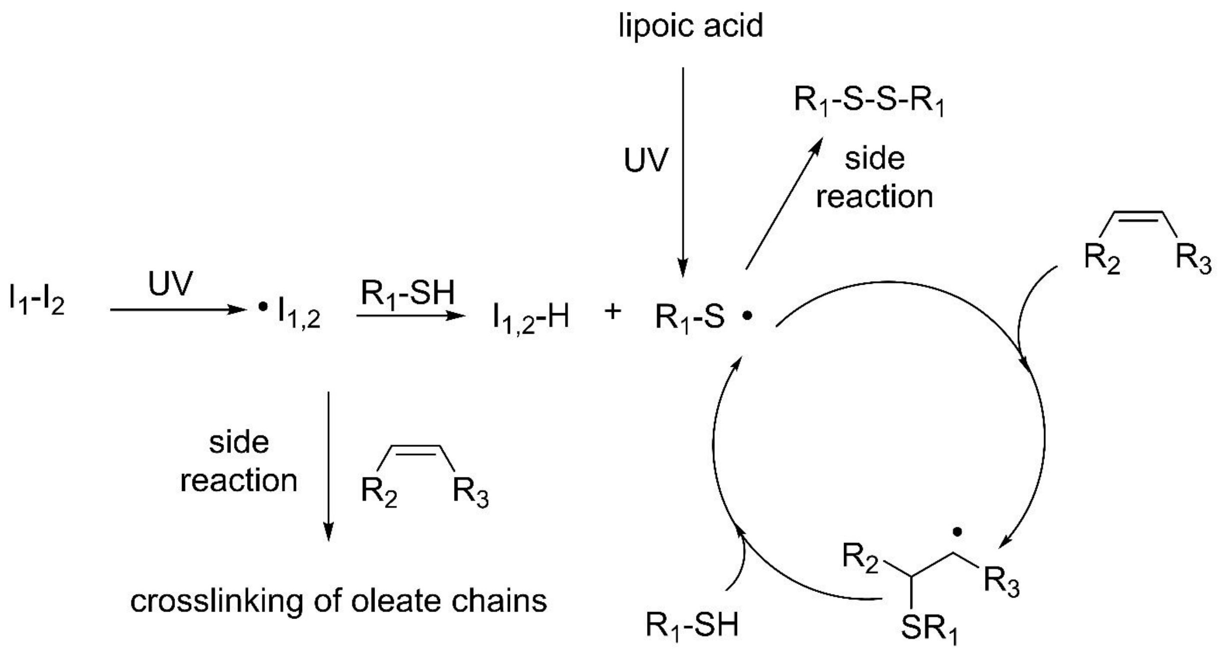

Scheme S1. The radical process of crosslinking starch oleate with both crosslinkers.

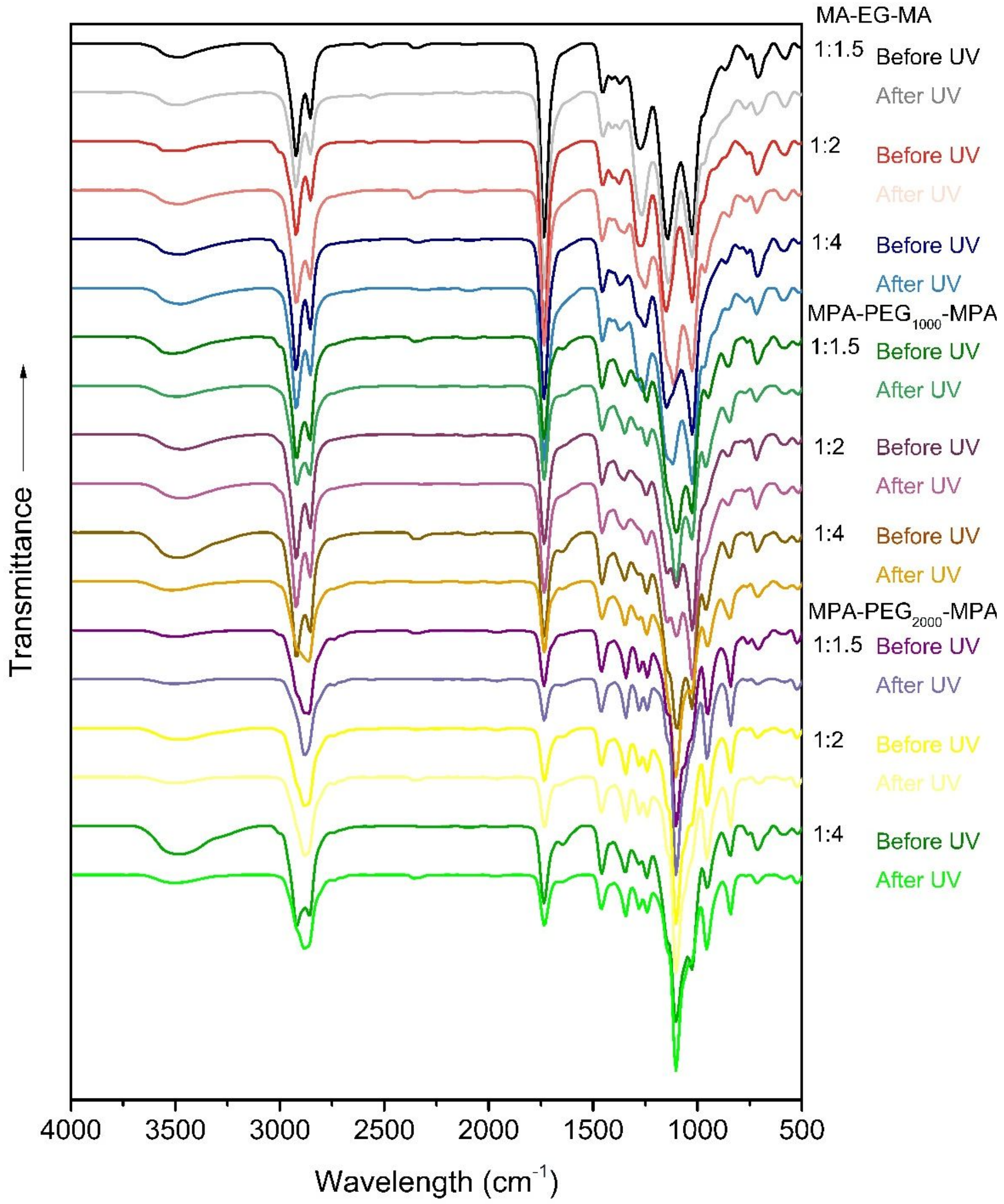

Figure S3. FTIR analysis of the SO films with M(P)A-based crosslinkers with ratio thiol:double bond of 1:1.5, 1:2, 1:4.

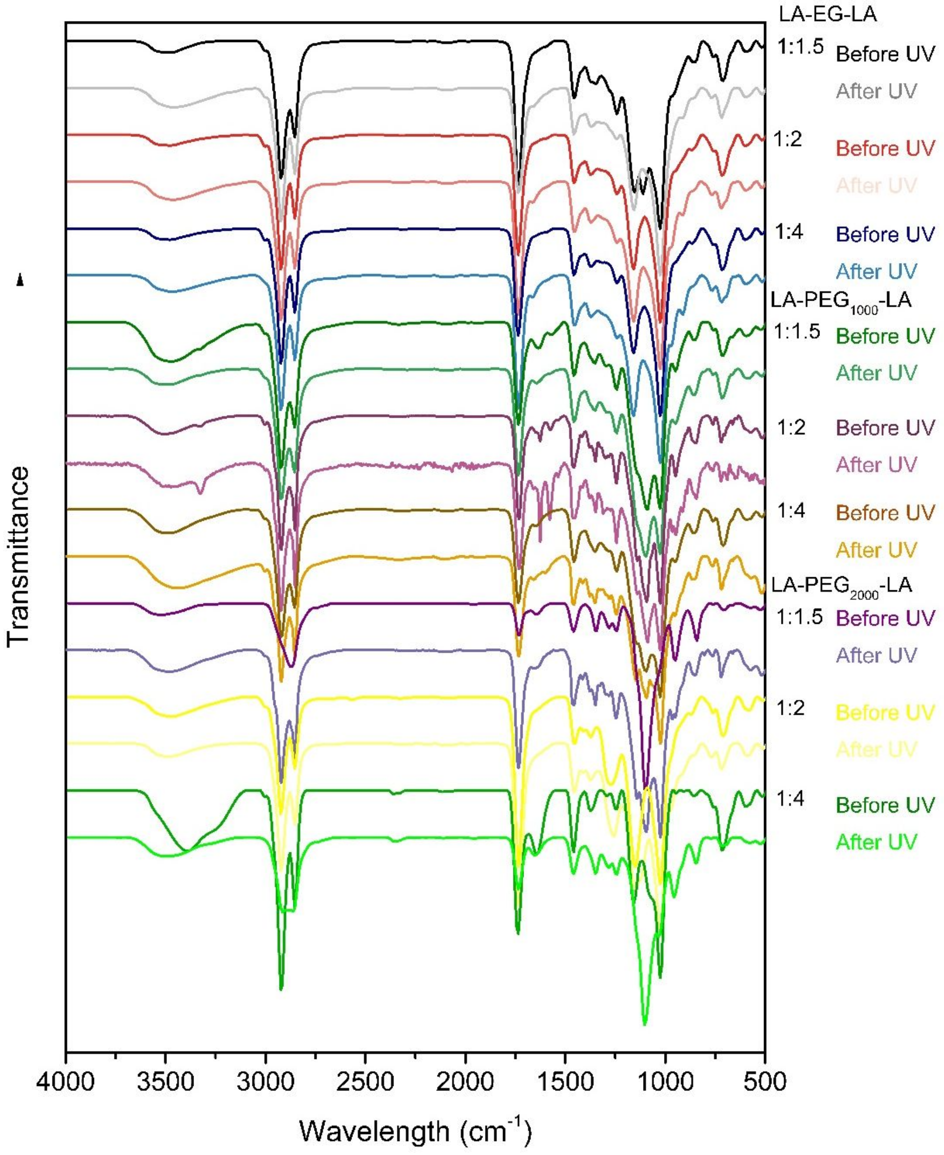

Figure S4. FT-IR analysis of the SO films with LA-based crosslinkers with ratio thiol:double bond of 1:1.5, 1:2, 1:4.

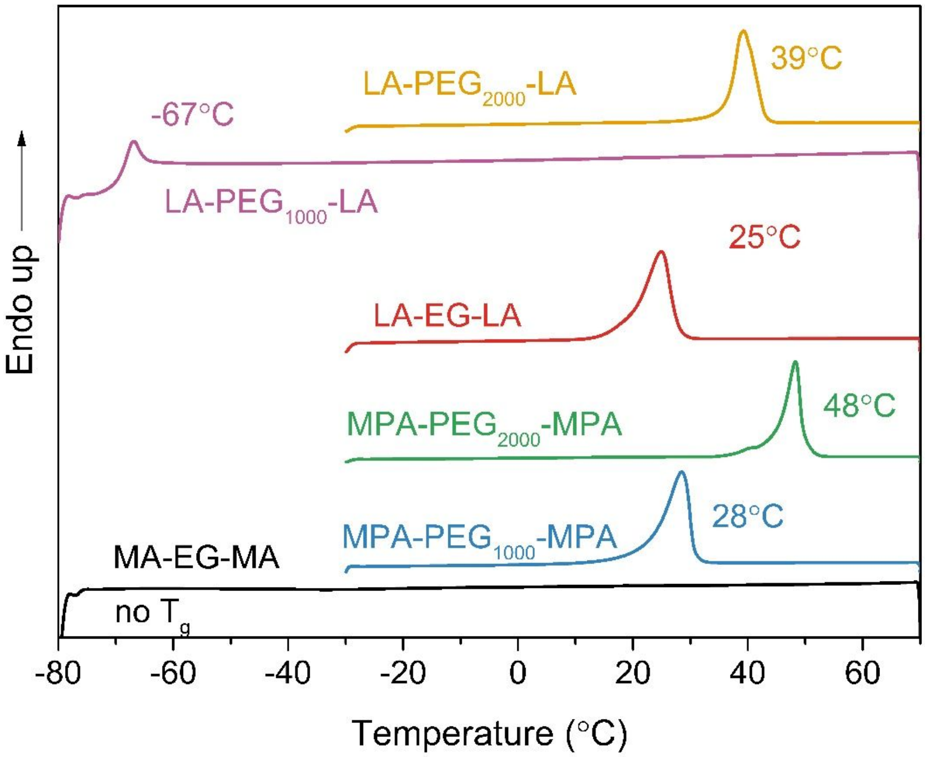

Figure S5. Melting points of the different crosslinkers used in this study

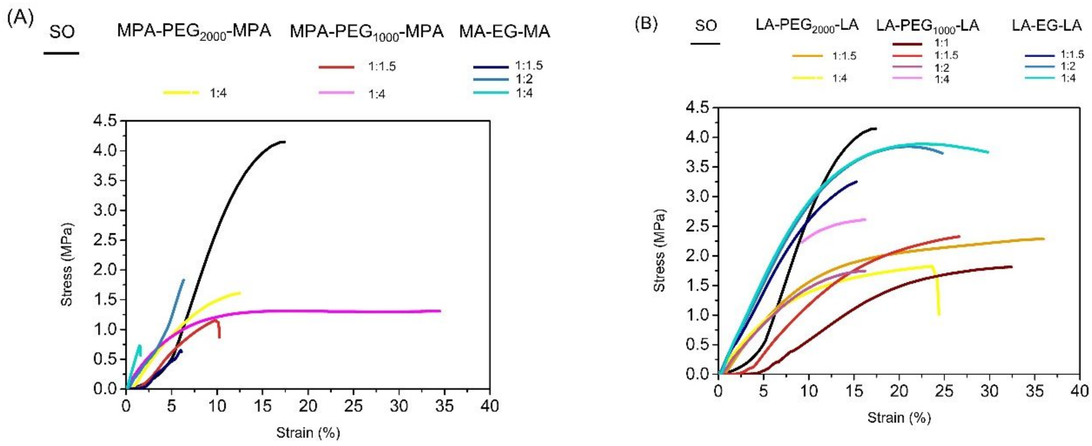

Figure S6. Stress strain cure of (A) films with MPA-based crosslinkers and (B) films with LA-based crosslinkers.
